# Custom-made asymmetric polyethylene liner to correct tibial component malposition in total knee arthroplasty — a case report

**DOI:** 10.1080/17453674.2018.1561384

**Published:** 2019-02-11

**Authors:** Andreas Kappel, Claes Sjørslev Blom, Anders El-Galaly

**Affiliations:** a Orthopaedic Research Unit, Aalborg University Hospital, Hobrovej, 9000 Aalborg;; b Department of Clinical Medicine, Aalborg University, Søndre Skovvej 15, 9000Aalborg, Denmark

A 56-year-old woman presented with knee pain, bow-leggedness, and instability following revision total knee arthroplasty 11 years previously.

A complex surgical history related to her right knee was revealed. At the age of 19, she suffered a midshaft tibial fracture treated non-operatively resulting in a sagittal bowing deformity. The anterior cruciate ligament was reconstructed at the age of 37 and a proximal bony correction using Ilizarov external fixation was done to correct recurvatum at the age of 38. In addition, 10 arthroscopic procedures were performed on the knee from the age of 20 to 34 years. A primary cemented TKA was performed at the age of 44 (NexGen CR, femur size C, tibia size 3, polyethylene 12 mm and patella size 29; Zimmer Biomet, Warsaw, IN, USA). Due to instability a partial revision was done 5 months later where the femoral component was brought distally and the polyethylene liner changed to LPS (NexGen LCCK femur size C, stem 12 × 100 mm, medial and lateral augments size 5mm, polyethylene size 14 LPS). However, pain, malalignment, and instability persisted.

Physical examination revealed a varus leg with varus thrust and lateral laxity of 5–10° in both extension and flexion, and limited knee hyperextension with flexion to 120°. There was no pathological medial or sagittal laxity, normal patellar tracking and no signs of malrotation. Radiographs revealed well-fixed components (Figure 1). Supplementary CT scan showed correct rotational placement of components. An EOS scan revealed coronal malposition of the tibial component with with mechanical tibiofemoral angulation of 9° varus (mechanical lateral distal femoral angle (mLDFA) = 91°, mechanical medial proximal tibial angle (mMPTA) = 82°) and a sagittal deformity of the tibia with posterior translation of the plateau and increased posterior slope (Figure 2).

**Figure 1.**
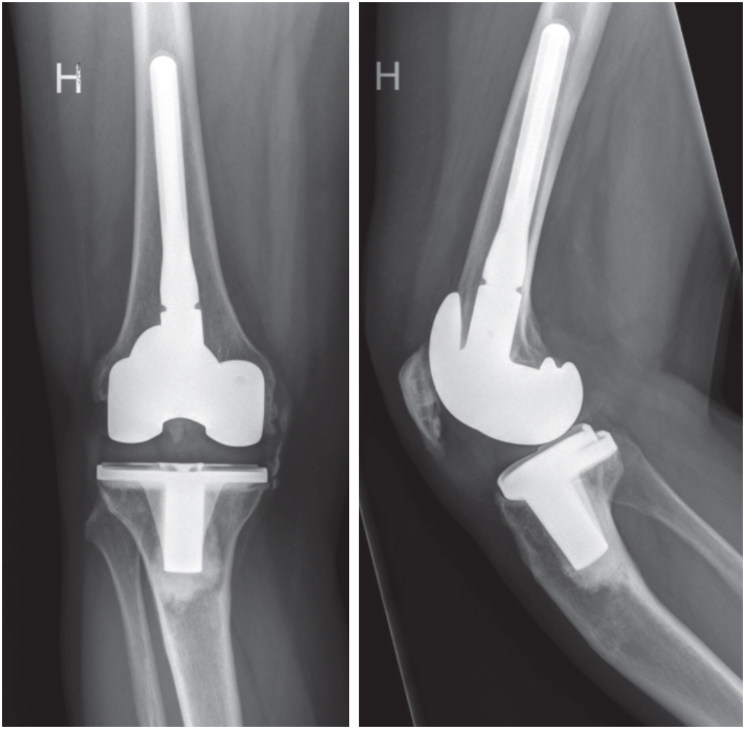
Before liner exchange.

**Figure 2.**
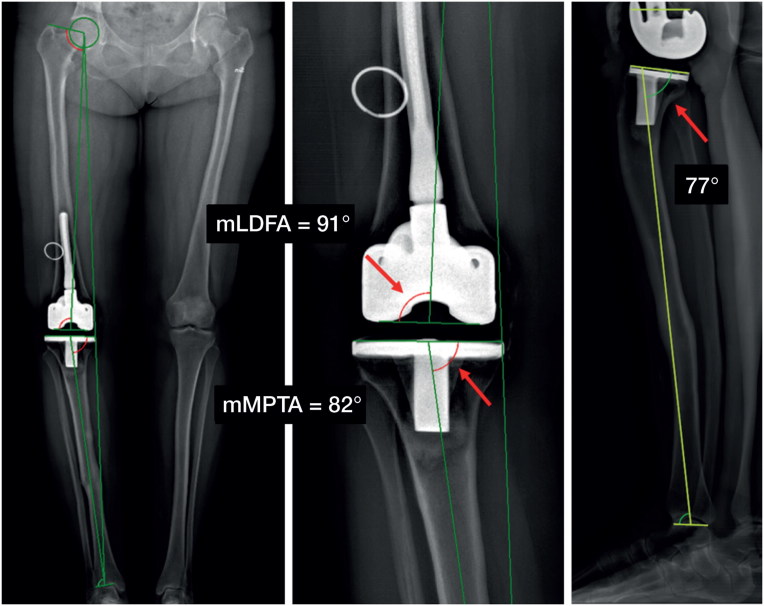
EOS scan before liner exchange.

Since only the tibial component was malpositioned and the soft-tissue envelope was intact, we therefore decided to correct the malalignment with a custom-made asymmetric polyethylene liner that was designed in cooperation with the manufacturer (Figure 3). The design incorporated a medial build-up of 6 millimeters to correct the 9° of varus and a slight posterior build-up of 3° to diminish the excessive slope of the tibial plateau and component.

**Figure 3.**
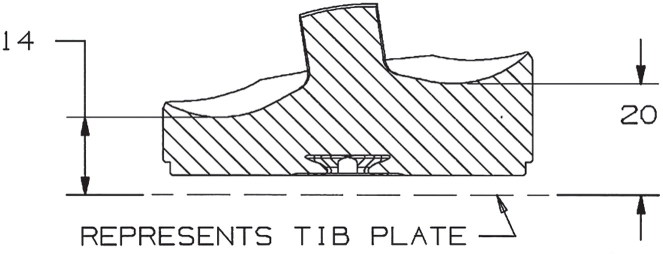
Custom liner construct.

The revision surgery was uneventful. Following moderate medial and posteromedial release the liner was inserted, and both alignment and stability was found to be satisfactory (Figure 4).

**Figure 4.**
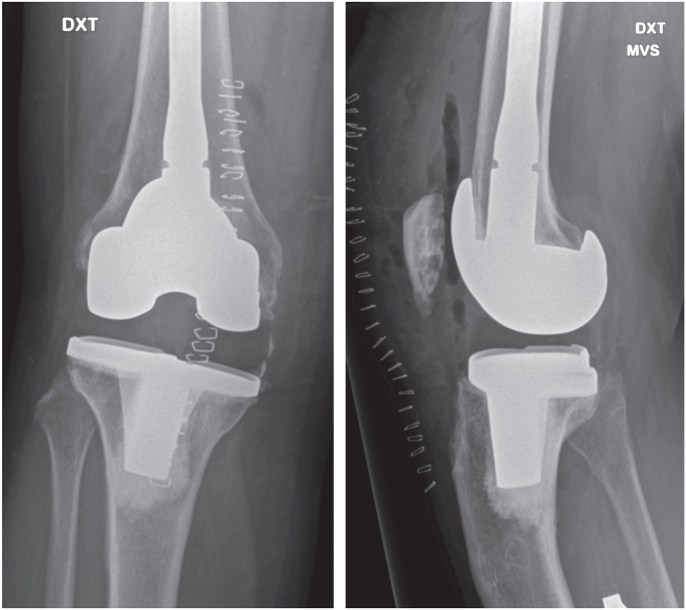
After liner exchange.

At 1-year follow-up the pain had decreased significantly and the patient had no complaints of malalignment or instability. Range of motion was from full extension to 120° flexion. We found no medial or sagittal laxity but still a lateral laxity of 5–10° in both extension and flexion. Outcome scores showed improvements from preoperative to 1-year follow-up: Oxford Knee Score (OKS) (from 12/48 to 31/48), EQ-5D-3L (from 0.3 to 0.7). The EOS scan at 1-year follow-up demonstrated neutral mechanical alignment (Figure 5).

**Figure 5.**
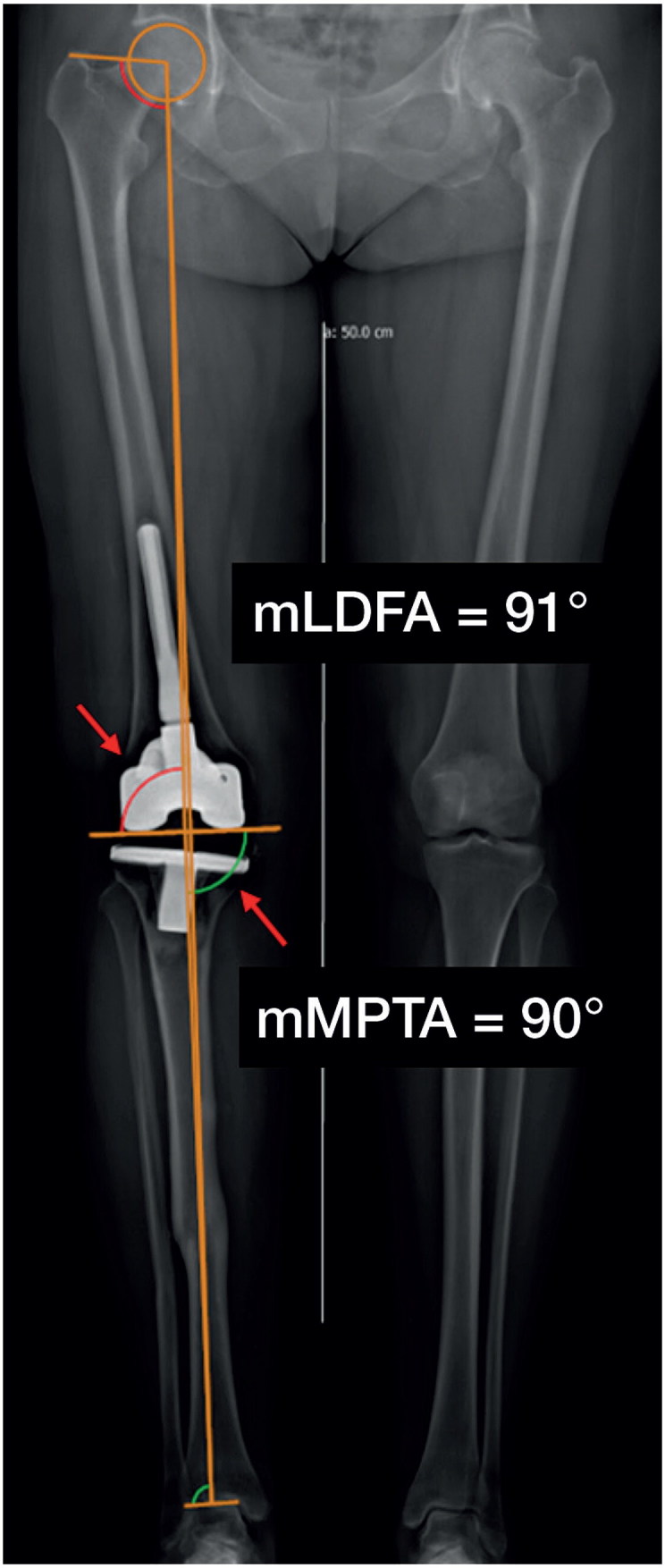
EOS scan after liner exchange.

## Discussion

Bony deformity might complicate both primary and revision TKA thus meticulous pre-surgical planning of both bony resection and choice of implant are advisable to secure the mechanical axis and stability of the joint (Xiao-Gang et al. 2012, Loures et al. 2018).

**Figure 6.**
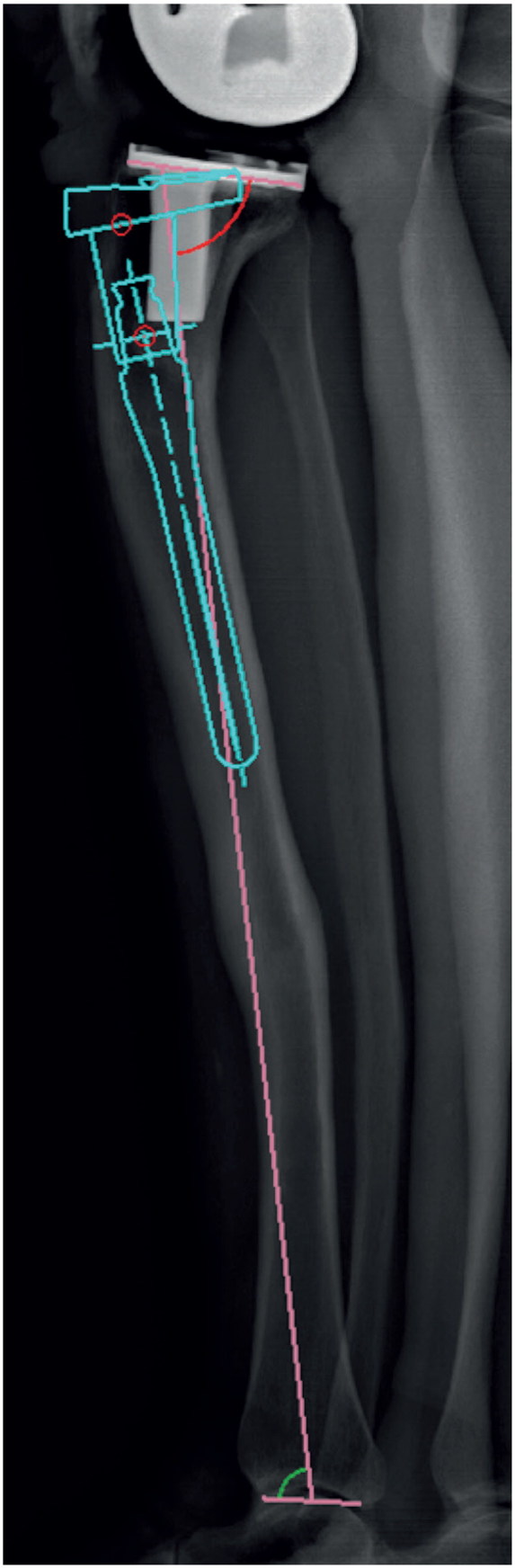
Templating tibia.

In this case, malpositioning of the tibial component caused varus malalignment. Tibial component revision would be the standard treatment to address this. However, this otherwise straightforward procedure was considered rather complicated, as a standard stemmed component, due to the posterior translation of the tibial plateau, would not fit the actual anatomy (Figure 6). We considered the use of a very short cemented stem, but due to the bony deformity the risk of repeated malpositioning and risk of difficulties in balancing the knee gave reason for concern. A hinged implant was less tempting due to the young age of the patient. Correction of the sagittal deformity with one or more osteotomies was also considered, but the complexity and high risk of complications caused concern. The decision to use a custom-made liner was preceded by thorough physical and radiological examination. Coronal mechanical alignment, sagittal alignment and component rotation was examined with EOS and CT scan. Coronal and sagittal malposition of the tibial component was evident while the femoral component was well placed and the soft-tissue envelope intact.

Changes in liner symmetries may affect alignment, soft-tissue tension, and ROM. The insertion of an asymmetric liner to restore mechanical alignment affects soft-tissue balance throughout the whole ROM and in our opinion requires that the femoral component is positioned absolutely correctly.

While the mechanical alignment is restored with the custom implant, asymmetric stresses are introduced to both the tibial bony fixation and the tibial component polyethylene locking mechanism. Whether these asymmetric stresses do affect longevity by increasing the risk of aseptic loosening or tibial backside wear might be a reason for concern (Rao et al. 2002, Gromov et al. 2014). Our patient had a satisfactory 1-year follow up with no radiographic loosening and improved patient-reported outcomes.

The use of a custom-made liner has been previously described by Sah et al. (2008) who used this technique to correct excessive slope of the tibial component in a complex primary TKA; however, in that case only sagittal correction was intended. To our knowledge, no previous reports have described the use of a custom liner to correct coronal or combined coronal and sagittal alignment.

In our opinion, the use of a custom-made polyethylene liner offers a simple alternative to more complex revision knee surgery with good short-term follow up in this case. However, the technique is an option only in isolated tibial malposition and is dependent on soft-tissue stability and/or level of constraint.
